# Normothermic regional perfusion surgical technique for the procurement of cardiac donors after circulatory death

**DOI:** 10.1016/j.xjtc.2022.01.016

**Published:** 2022-01-21

**Authors:** Amy G. Fiedler, Stephen DeVries, Clarisa Czekajlo, Jason W. Smith

**Affiliations:** Division of Cardiothoracic Surgery, Department of Surgery, University of Wisconsin, Madison, Wis

**Figure:**
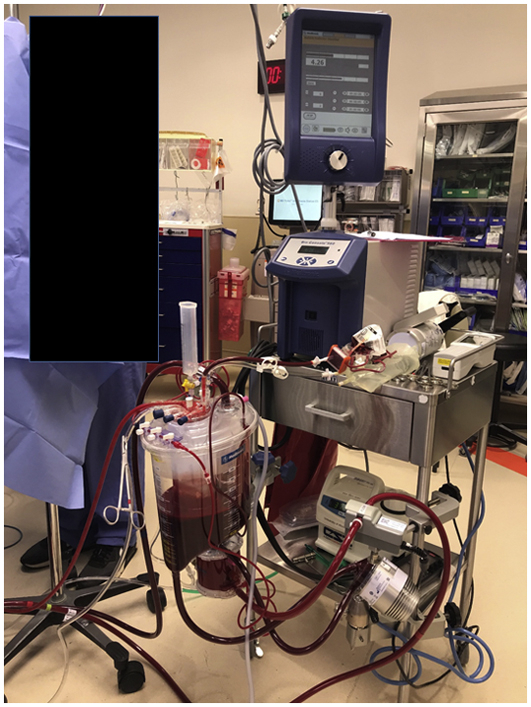
VA-ECMO NRP circuit used in a University of Wisconsin DCD heart procurement.

**Central Message**

DCD heart transplant has not been routinely used. We describe a low-cost, reproducible method for DCD heart procurement using NRP allowing for distant procurement with cold static storage.

See Commentary on page 116.

Donation after circulatory death (DCD) has been used outside of the United States to expand the cardiac donor pool with good results. Currently, recovery methods include the use of the Organ Care System (OCS; TransMedics) or normothermic regional perfusion (NRP).^1^, ^2^, ^3^ Here, we describe The University of Wisconsin (UW) methodology of DCD organ selection, surgical technique, intraoperative graft evaluation, and ultimate procurement of a DCD heart using NRP. Our technique demonstrates the capacity to procure DCD hearts from distant centers using NRP and cold static storage in a reliable fashion.

The institutional review board or equivalent ethics committee at UW did not approve this study, as it falls outside the scope of necessary approval. Patient written consent for the publication of the study was not received, as this is retrospective analysis of deceased individuals.

## Technique

### Premortem

Donor selection and premortem management are outside the scope of this review. Depending on hospital policies, life-sustaining treatments are withdrawn from the donor in either the intensive care unit or the operating room. If the donor dies within 30 minutes from agonal (defined as oxygen saturation less than 70% and blood pressure less than 50 systolic) a mandatory hands-off period is undertaken to prevent autoresuscitation. The donor is declared dead by cardiorespiratory examination performed by a physician. The procurement may now begin.

### Venoarterial Extracorporeal Membrane Oxygenation (VA-ECMO) Circuit Configuration

**Figure 1:**
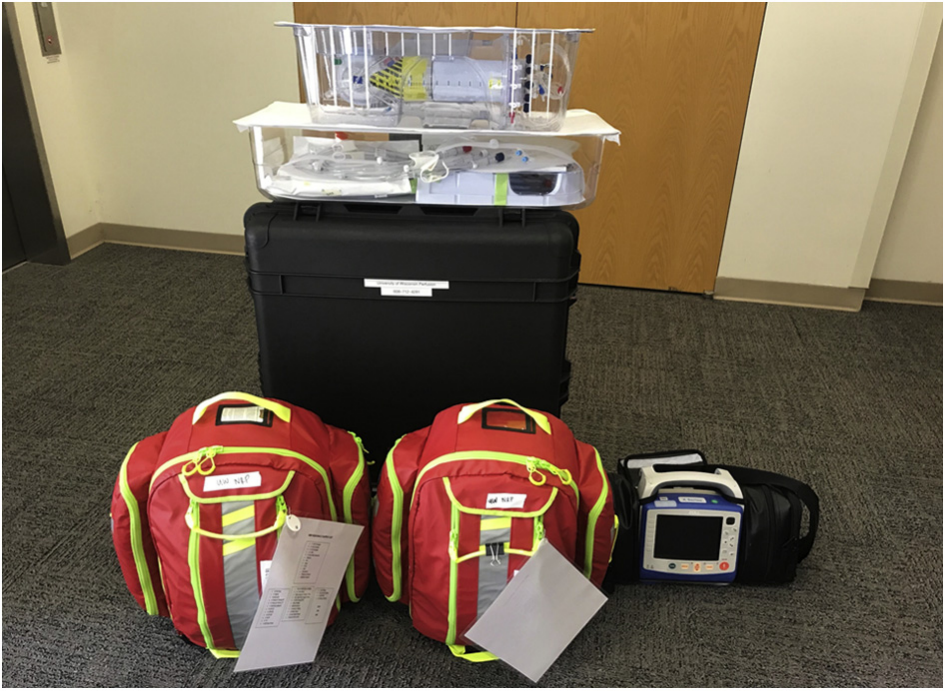
The University of Wisconsin venoarterial extracorporeal membrane oxygenation normothermic regional perfusion circuit disassembled into portable travel packs.

Our portable VA-ECMO NRP circuit consists of a Biomedicus 560 Centrifugal Pump (Medtronic), Affinity Fusion oxygenation system with cardiotomy venous reservoir (Medtronic), and Affinity CP centrifugal pump head (Medtronic). Two quarter-inch tubing lines are connected from the sterile field to the cardiotomy reservoir to serve as field suction. Field and venous drainage are augmented as needed using vacuum assist. The prime volume consists of 1 L of PlasmaLyte (Baxter International, Inc) with normothermia maintained using a Micro-Temp LT portable heater cooler (Gentherm Medical; Figure 1). For full details regarding the UW DCD-NRP run bag, please reference the Online Data Supplement.

### Chest Entry and Cannulation

Following declaration of death, an incision is made to the level of the sternum. The sternum is then expeditiously split with a sternal saw. A sternal retractor is placed, and the pericardium is opened sharply with scissors. Extreme care must be taken to prevent inadvertent injury to the innominate vein at this time.

With the use of an 11 blade knife, the right atrium is opened and an Edwards Lifesciences 36/46 Fr dual-stage venous cannula (Edwards Lifesciences) is introduced. Vacuum is then applied to the VA-ECMO NRP circuit to assist with cardiac decompression. A silk tie is passed around the insertion point of the cannula including atrial tissue for security and to prevent air locking.

The heart is retracted left ward by the assistant and the aortic arch dissection is carried out sharply. Once the brachiocephalic vessels are identified, the innominate artery and left carotid artery are clamped with straight vascular clamps to exclude the cerebral circulation.

Following this, the adventitia is cleared from the ascending aorta, and with an 11 blade knife a stab incision is made for the aortic cannula. A Medtronic 20Fr aortic cannula is then introduced and connected in a wet-to-wet fashion to the arterial limb of the VA-ECMO NRP circuit. Clamps are released and the donor is placed on NRP. PROLENE purse-string sutures (Ethicon) are then placed around the aortic and venous cannulae and secured with rummell tourniquet.

### Cardiac Assessment and Management

Once the donor has been successfully placed on NRP support, flow is titrated to maintain a cardiac index of 2 or greater. A Bentley needle is inserted directly into the ascending aorta to function as a pressure measurement line and a delivery system for cold cardioplegia. The patient is then reintubated with low tidal volume ventilation to prevent elevating the pulmonary vascular resistance secondary to atelectatic lungs. Vasopressors are titrated for a mean arterial pressure goal of greater than 60 mm Hg and managed per the organ-procurement organization or anesthesia staff.

We maintain NRP perfusion for 45 minutes. During this time, the chest and abdominal teams perform standard procurement dissection. After 45 minutes of support, the donor is weaned gradually off of NRP and the heart is visually assessed for function by a skilled attending cardiac surgeon (Video 1). We remain off NRP for 2 minutes to watch the heart under true loading conditions. Should the heart appear suitable for transplantation, full NRP flow is then re-established, and the implanting surgeon is instructed that the recipient operation should commence.

**Video 1:**
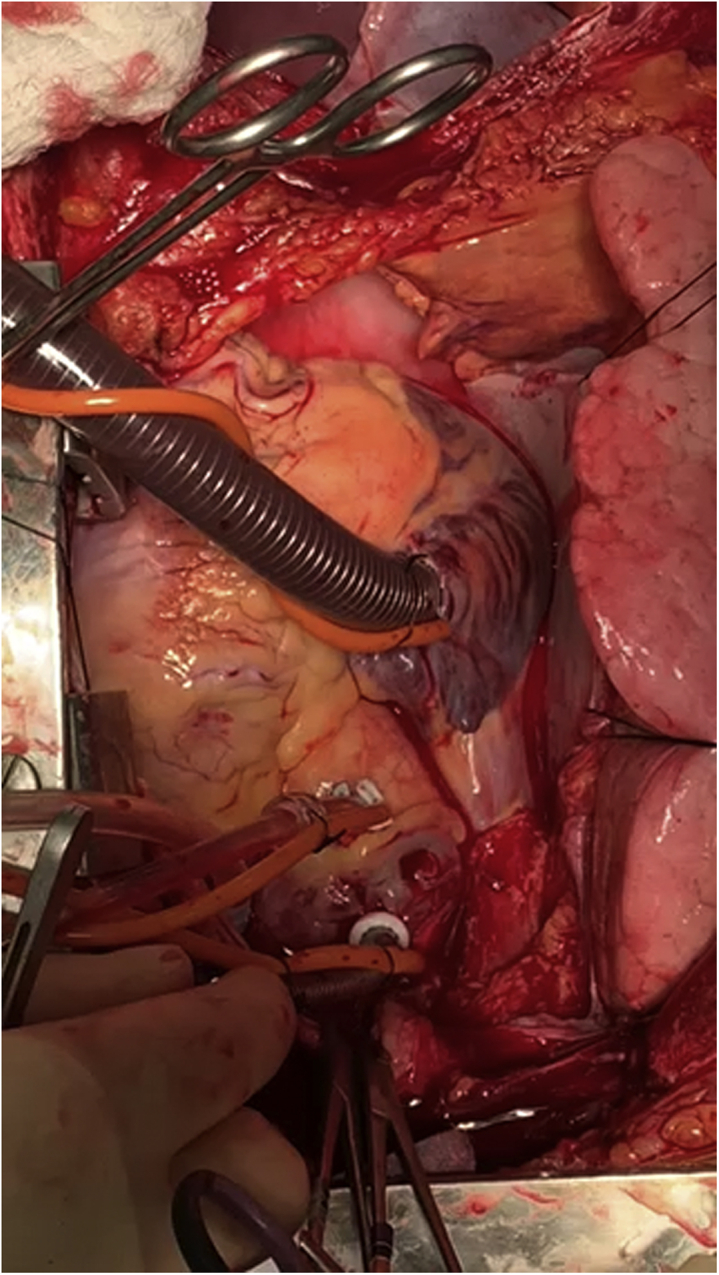
Donor graft cannulated for VA-ECMO NRP in the assessment period off support following a 45-minute period of reperfusion. Video available at: https://www.jtcvs.org/article/S2666-2507(22)00046-3/fulltext.

Procurement of the heart and additional donor organs for transplantation is then undertaken in the standard fashion. The heart is evaluated and packaged on the back table and returned to the home institution via cold static storage.

### Preliminary Outcomes

Since the inception of our DCD NRP program, we have procured and subsequently transplanted 10 hearts via the protocol described in this manuscript. There has only been one donor organ that was placed on NRP, observed for approximately 45 minutes, and was deemed unsuitable for full procurement and subsequent transplantation. During this procurement process, we kept the heart on NRP until the abdominal procurement team was ready to clamp and flush to continue to preserve the abdominal organs. We have not procured a heart using NRP onto an OCS; all of our transplants have been stored with cold static storage following recovery.

A manuscript has been submitted regarding our initial outcomes using the aforementioned protocol and process. In short, the short-term outcomes have been found to be noninferior when compared with our institutional experience with brain dead donors as well as our experience in the TransMedics OCS consortium evaluating the outcomes of DCD hearts procured and maintained on OCS.

## Comment

**Figure 2:**
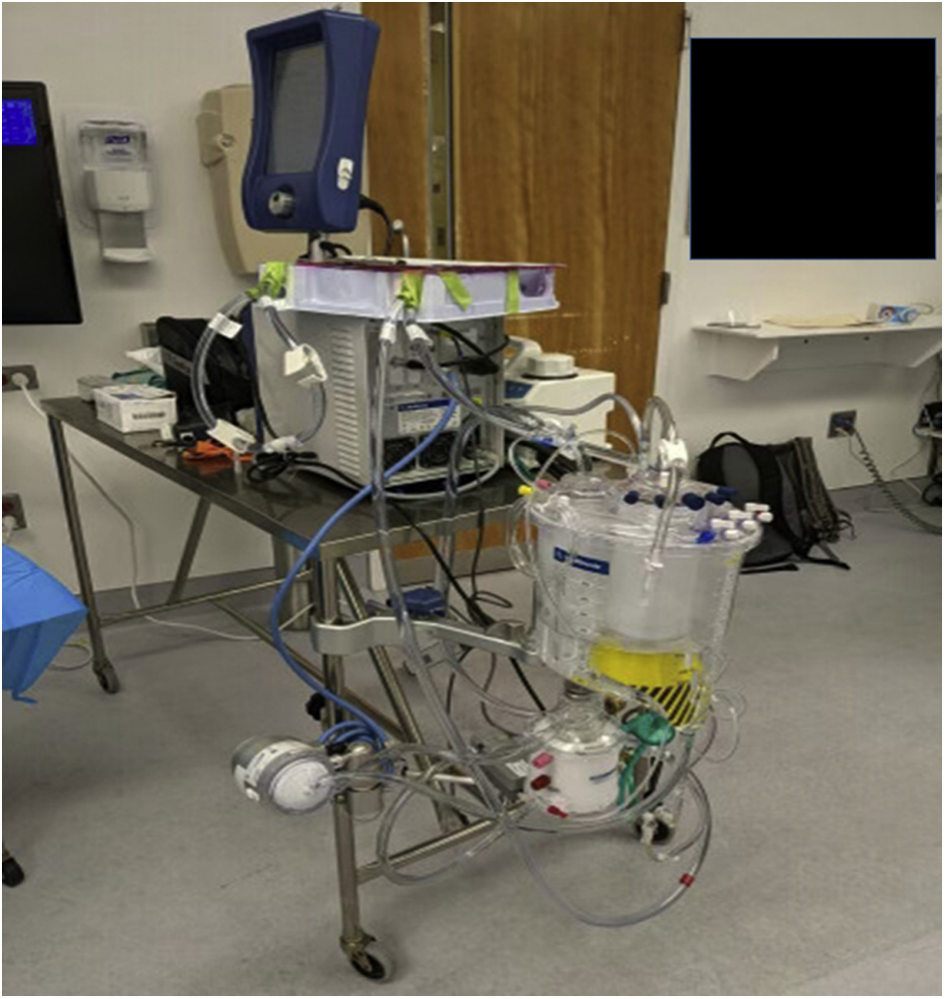
The University of Wisconsin venoarterial extracorporeal membrane oxygenation normothermic regional perfusion circuit primed and ready for use.

The UW technique for DCD-NRP heart transplantation with cold static storage is portable, reproducible, and low-cost, allowing for expansion of DCD cardiac transplantation even at distant donor locations (Figure 2).

## Supplementary Data

Video 1 Donor graft cannulated for VA-ECMO NRP in the assessment period off support following a 45-minute period of reperfusion. Video available at: https://www.jtcvs.org/article/S2666-2507(22)00046-3/fulltext.
