# Supplementary material for: Normothermic regional perfusion surgical technique for the procurement of cardiac donors after circulatory death
Source: JTCVS Tech. 2022 Jan 21;12:113–5. doi: 10.1016/j.xjtc.2022.01.016 (PMC8987395; doi:10.1016/j.xjtc.2022.01.016)
Supplement: Online Data Supplement [file mmc2.docx]

**Supplementary Appendix:**

Contents of the run-bag for UW NRP-DCD Heart Procurement

- - - 1. 6 dark blue pacing (V Wires)
      2. 4 red pacing (A Wires)
      3. 4 white pacing cables
      4. 4 blue pacing cables
      5. 1 ECMO drug tray (10,000 heparin x5, 50meq bicarb, 25% albumin 50msl x2, 1g calcium)
      6. 4 AA batteries for pacer
      7. 4 9v batteries for istat
      8. Syringes (1-10CC)/ 18G Needles x 10 of each
      9. 10 - 4.0 Prolene SH-1 30”
      10. 10 - 2.0 Ethibond excel SH 36”
      11. 6 - 3.0 Prolene SH-1 30”
      12. 2- permahandsilk 60”
      13. 2- umbilical tape
      14. 2- fogarty softjaw insert set 86cm
      15. 2- 0 Vicryl CT 1 27” (8 per packet)
      16. Non- Sterile scissors
      17. 20- 25G needles
      18. 15- 22G needles
      19. 4- pledget packets
      20. 2- 1L sterile water
      21. 6- 1L plasmalyte
      22. 20- zip ties
      23. ECMO Bridge Line
      24. 2- ⅜ tubing
      25. 2- blue chucks
      26. 2- EOPA 3D arterial cannula
      27. 1- 18 arterial cannula without wire
      28. 2- 36/46 MC2
      29. 1- 32/40 MC2
      30. 2- aseptos (cath tip syringe)
      31. 2- bulb syringes
      32. 2- 14 g aortic root needles
      33. 2 - blood filters
      34. 2- cook needles
      35. 3- 6 fr sheaths
      36. 2- Quick Prime Lines
      37. Pacer box
      38. 4- 10 blades
      39. 20- alcohol wipes
      40. 8- non-sterile tubing clamps
      41. 2- ultrasound covers + gel
      42. 3- pack of 4 red tourniquet snares
      43. 4- ⅜-⅜-⅜ wyes
      44. 4- ⅜ straight connector
      45. 3- ⅜-½ connector
      46. 2- quick prime line
      47. 4- 5% albumins 250mls
      48. 4- stopcocks
      49. 4-pressure monitoring lines
      50. 1 - custom ecmo tubing pack
      51. 1- medtronic affinity reservoir + oxygenator
      52. 2centrifugal pump head medtronic affinity
      53. 2- Red Biohazard Bags
      54. Temperature Probe
      55. 16L High Flow Oxygen Meter + Green Tubing
      56. Zip Tie gun
      57. Internal Paddles (compatible with our Zoll Defibrillator)
